# Point-of-Care Ultrasound (POCUS) in the Field of Diabetology

**DOI:** 10.1155/2021/8857016

**Published:** 2021-03-08

**Authors:** X. Vandemergel

**Affiliations:** Department of Endocrinology and Diabetology, EpiCURA, Belgium

## Abstract

Ultrasound is increasingly used in daily clinical practice to improve the efficiency of the clinical examination. In this article, we reviewed its various possible uses in the field of diabetology. The ultrasonic evaluation of the carotid arteries (plaques and intima media thickness) allows improving the assessment of the cardiovascular risk. Steatosis can be detected relatively easily on liver ultrasound. Ultrasound also allows a more sensitive detection of lipohypertrophy resulting in glycemic fluctuations and thus increasing the risk of hypoglycemia than the clinical examination. Finally, muscle ultrasound appears to be a promising tool to assess the nutritional status and its consequences (e.g., falls).

## 1. Introduction

Diabetes is a systemic disease. Its global prevalence is estimated to be 9.3% (463 million people), rising to 10.2% by 2030 [1], being a major public health issue. Patients with type 1 or type 2 (T2D) diabetes (DP) are at risk for micro- and macrovascular complications [2]. The management of DP is therefore multifactorial and requires, in addition to sometimes restrictive treatments, regular monitoring and screening examination (fundus examination, electrocardiogram, detection of microalbuminuria, dental follow-up, and vaccination). The multiplicity of examinations regularly decreases the patient's compliance. For example, in the study by Murchison et al., the rate of adherence to the follow-up recommendations in the context of ocular examinations was disappointingly low regardless of the age and ethnic group, ranging from 35% to 65% depending on the severity of retinopathy [3]. Moreover, epidemiological evidence suggests that T2D patients are at significantly higher risk for many types of cancer, including liver, pancreas, endometrium, breast, and colorectal cancers [4, 5]. This association requires a careful clinical examination of DP and a close follow-up with appropriate cancer screening as recommended for all individuals depending on their age and sex. While technological advances have been made in recent years in terms of self-monitoring or treatment (smartphone applications, subcutaneous glucose monitoring, and insulin pumps) [6–8], the tools used during the quarterly or semiannual consultations by diabetologists have not changed much. Indeed, a stethoscope, reflex hammer, tuning fork, and monofilament are still the basis for the examination. While the clinical examination remains a fundamental step in the clinical approach, it should be kept in mind that it does not significantly contribute to the diagnosis, even when a stethoscope is used. In a prospective study conducted in 80 medical outpatients with new or previously undiagnosed conditions published in 1992, Peterson et al. [9] have shown that the anamnesis alone allowed making a diagnosis in 80% of cases while the physical examination alone allowed making a diagnosis in only 12% of cases. In a similar study conducted in patients with cardiovascular diseases, Sandler [10] had reported that 56%, 17%, and 23% of diagnoses were made based on patient history, physical examination, and laboratory tests, respectively. Tools allowing improving the performance of our clinical examinations are thus needed.

### 1.1. Introduction of Ultrasound in Medicine and Advent of POCUS

Cardiologists have integrated ultrasound (US) in their daily practice since 1954 [11], followed four years later by gynecologists [12], and it took until 1979 for the first publication of a radiological study assessing the interest of US in patients with right upper quadrant pain and gallstones [13]. In recent years, a new generation of clinicians have implemented the use of US in their daily practice such as in emergency medicine (as well as in war and disaster medicine) and in intensive care units [14–16] and more recently in other specialties such as internal general medicine [17] and rheumatology [18].

Point-of-care ultrasonography (POCUS), i.e., ultrasonography performed and interpreted by the clinician at the bedside or in the ambulatory setting, has emerged thanks to multiple factors including technology improvement and the fact that the US equipment has become more compact, with an enhanced image quality, and is equipped with software to facilitate result interpretation. It offers several advantages: it is noninvasive and nonirradiating and is performed at the bedside and it allows saving time [17].

Furthermore, trained and young physicians are significantly more competent and attentive when using US. In a study, first-year medical students who used POCUS obtained better results in identifying heart abnormalities than board-certified cardiologists who used a bedside cardiovascular physical examination, with, respectively, 75% and 49% of cases identified by students and cardiologists (using only a stethoscope) [19]. In another study, the authors have shown that medical students who used POCUS estimated more accurately the liver size than board-certified internists who performed a physical examination [20].

Only a few studies have investigated the interest of POCUS in diabetology. However, as we will see, diabetology is a specialty in which POCUS could find a special place to improve the diagnostic efficiency and to simplify the management and decision-making.

### 1.2. Some Technical Considerations

US is defined as a frequency higher than the upper audible limit of human hearing, i.e., greater than 20,000 Hz (20 kHz). The frequency of diagnostic US is in the range of 1 million hertz (MHz). Lower-frequency US has a better penetration, but a lower resolution. Higher-frequency US provides higher quality images, but it does not allow visualizing the deep structures. A typical transabdominal or cardiac probe has a frequency in the range of 2-5 MHz, whereas probes used for the superficial structures such as the carotids, thyroid, or muscles have a frequency in the range of 10-15 MHz. Three modes may be used for image analysis: (1) the B mode (real-time analysis of a structure), (2) the M mode which is a diagnostic US presentation of the temporal changes in echoes in which the depth of echo-producing interfaces is displayed along one axis and time (*T*) is displayed along the second axis and the motion (*M*) of the interfaces is recorded toward and away from the transducer, and finally (3) the Doppler mode to analyze the blood flow.

## 2. Assessment of the Cardiovascular Risk

**Figure 1:**
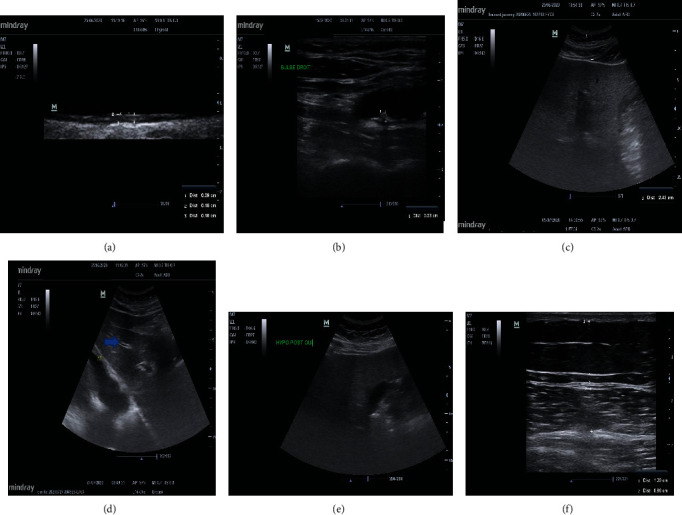
(a) Normal carotid artery ultrasonography. High-frequency probe. The intima media thickness is measured at three points. (b) Visualization of a plaque of 0.23 mm thickness located near the carotid bifurcation. High-frequency probe. (c) Liver echography with determination of the skin capsular distance (2.43 cm). Low-frequency curvilinear probe. (d) Normal liver echography showing the portal veins (horizontal arrow), the diaphragm, and a homogeneous parenchyma. Low-frequency curvilinear probe. (e) Nonalcoholic steatohepatitis. This picture shows a rapid ultrasound attenuation. The vessels are attenuated, and the posterior liver is not seen. (f) Measurement of the rectus femoris (1) and vastus intermedius (2) thickness. High-frequency linear probe.

DP are at high risk of cardiovascular diseases (CVD) [21]. Their risk of incident coronary heart disease or ischemic stroke is increased by 2-4 and their risk of mortality by 1.5-3.6 compared to nondiabetic subjects (NDS) [22]. T2D is also a major risk factor for heart failure, peripheral arterial insufficiency, and microvascular complications, affecting patients' quality of life and life expectancy. In DP, the life expectancy is estimated to be reduced by 4 to 8 years compared to that in NDS [23]. Traditional risk factors such as aging, hypertension, hyperlipidemia, or smoking have been shown to only moderately refine the CV risk [24, 25], and the use of calculators is needed to improve the risk prediction. However, at least 110 different CV risk score calculators are currently used and 45 exclusively in DP [26]. Due to differences in databases and diverse mathematical algorithms and to the different combinations of CVD endpoints, there is considerable variability in the scores obtained. It is important to keep in mind that the validation of these scores is limited to the characteristics of the studied population. In contrast, invasive procedures such as coronary angiography or coronary computed tomography angiography can be used to determine the presence and severity of coronary artery disease (CAD) but with potential adverse effects and high costs. Determining more precisely the CV risks of DP is crucial to identify which patients are likely to benefit from preventive treatment. For example, in the Ascend trial, administering low-dose aspirin to all DP improved CVD prevention while it increased the risk of hemorrhage [27]. Therefore, noninvasive and inexpensive indices of subclinical and silent atherosclerosis with more than moderate predictive capacity are required. Carotid ultrasonography (CU) is a promising tool to achieve it. CU may show atherosclerotic changes including intima media thickening (IMT or carotid intima media thickness (CIMT)) and plaque formation which allows analyzing the plaque structure, stenosis, or vessel occlusion. The CIMT is a well-described surrogate marker for CVD. The carotids are evaluated using a high-frequency (10-14 MHz) linear probe. In B mode, the carotid wall is visualized as three layers (Figure 1(a)). The two layers closer to the vascular lumen are defined as the “intima media” complex, and the thickness of the intima-media complex is defined as the CIMT [28]. The CIMT is classically measured near a carotid bifurcation, and US devices equipped with automatic IMT measurement software have recently become widely used to reduce interexaminer errors and the examination duration [28]. Plaques (Figure 1(b)) are defined as a focal wall thickening > 50% (or 0.5 mm) of the surrounding IMT or a CIMT > 1.5 mm [29]. The plaques are assessed based on their echogenicity, heterogeneity, and structure. The plaques may be characterized by their presence or absence, location, thickness, number, irregularity (smooth, irregular, or ulcerated), and echodensity (echolucent or echogenic) [29]. Many studies have shown that CU may be used to measure coronary atherosclerosis and myocardial ischemia [30, 31]. In a study by Akazama et al. conducted in 322 DP, 92% of patients with CAD had plaques compared to 54% of patients without CAD [32]. In the ARIC trial, Nambi et al. have reported that the prediction of the CAD risk could be improved by adding information on the CIMT and plaques to traditional risk factors [33]. In their study, 10% of patients had diabetes. When the CIMT was >75^th^ percentile and plaques were present in men, a dramatic elevation of the number of CV events was observed. Indeed, the adjusted incidence rate of coronary heart disease per 1000 person years was 24.7 in patients with a CIMT >75^th^ percentile and plaque versus 7.2 in patients with a CIMT <25^th^ percentile without plaque. They have also shown that taking into account the presence of plaques in addition to the CIMT significantly improved the risk of reclassification by 9.9% in the overall population and by 21.7% in the intermediate-risk group. It should be noted that a higher number of subjects were reclassified into a lower-risk group than into a higher-risk group. Regardless of the CIMT, the presence of plaques was associated with a higher incidence of coronary heart disease. The US assessment of carotid plaques has been shown to have a higher diagnostic accuracy than the CIMT for predicting future myocardial infarction. In addition, the absence of carotid plaque was more reassuring, with a low 10-year rate of myocardial infarction (4.0%). In another study [34], the authors have shown that after a 10-year follow-up, based on the US assessment of the carotid arteries, a first clinical event was identified in 3% of subjects with an initially normal US examination, in 32% of patients with IMT thickness, and in 62% of patients with an asymptomatic carotid plaque. In DP without apparent CVD, Irie et al. [35] have also shown that a maximum IMT was significantly associated with the presence of coronary artery stenosis.

Plaque features are also a valuable tool to assess the CV risk. The presence of a lipid-rich core, calcification, and ulceration is associated with a higher risk. In a large study including 582 DP who underwent CU, Vigili de Kreutzenberg et al. have found a prevalence of plaques of 82%. The plaque was echolucent in 16% of cases, heterogeneous in 43% of cases, and echogenic in 22% of cases while 19% of patients had no plaque. The presence of a plaque was associated with incident major CV events with a hazard ratio varying depending on the plaque features (1.97 (0.93-3.44) for echolucent plaques, 3.1 (2.09-4.23) for heterogeneous plaques, and 3.71 (2.09-5.59) for echogenic plaques) [36].

Therefore, CU appears to be a promising tool to assess the CV risk, but further studies are needed to validate the previous findings, particularly in the diabetic population.

### 2.1. Steatohepatitis

Nonalcoholic fatty liver disease (NAFLD) and T2D are common disorders that often coexist and can act synergistically to drive adverse outcomes. The reported prevalence of NAFLD in DP ranges from 29.6% to 87.1% [37, 38]. The presence of both NAFLD and T2D increases the risk for developing complications of diabetes (including both macro- and microvascular complications) as well as the risk of experiencing a more severe form of NAFLD, including cirrhosis, hepatocellular carcinoma, and death. Advanced fibrosis has been reported in 5-7% of asymptomatic subjects with T2D [39]. The alanine aminotransferase (ALT) level alone is not sufficient to rule out the presence of hepatic steatosis [40]. In a study published in 2019, Gawrieh et al. have evaluated 534 adults with biopsy-proven NAFLD and ALT and aspartate aminotransferase (AST) levels < 40 U/L within 3 months of their liver biopsy. The prevalence of stage F2-F3 nonalcoholic steatohepatitis (NASH) and cirrhosis was 19% and 7%, respectively. Detecting, assessing, and treating NAFLD in DP are thus required.

A meta-analysis of 49 studies including 4720 patients has found that the sensitivity and specificity of US were 85% and 94%, respectively, when using liver biopsy as the gold standard [41]. Different findings have been found in NAFLD: a hyperechoic texture or a bright liver due to a diffuse fatty infiltration and parenchyma heterogeneity, bright hepatic echoes, an increased hepatorenal echogenicity, a vascular blurring of the portal or hepatic vein (Figure 1(e)), and a rapid attenuation of the image within 4-5 cm of depth making deeper structures difficult to appreciate. The liver fills the entire field with no visible edges (considered helpful but not necessary for the diagnosis) [42]. When steatosis affects >30% of the liver, a bright liver echo pattern is present in 89% of cases [43]. In this study, the sensitivity, specificity, and positive and negative predictive values of a bright liver echo pattern for steatosis were 64%, 97%, 96%, and 65%, respectively, and in the subgroup of patients with steatosis of ≥30%, these values were 91%, 93%, 89%, and 94%, respectively.

The subcutaneous tissue thickness (Figure 1(c)), mainly made of fatty tissue, measured as the distance between the skin surface and the liver surface, also called the “skin capsular distance” (SCD), has been shown to be another characteristic sonographic finding that can be easily assessed. It has been shown that the SCD may be used in patients to diagnose NASH. In 101 patients with NASH, Shen et al. have shown that 70% of patients had a SCD > 25 mm. When the SCD was <25 mm, only 20% of patients had NASH [44]. Riley et al. [45] have found in a comparative study that NAFLD patients had a thicker subcutaneous tissue, with a mean thickness of 25.6 ± 5.6 mm. In comparison, non-NAFLD patients had a mean subcutaneous tissue thickness of 19.5 ± 5.2 mm (*p* < 0.001). In addition, NAFLD was unlikely when the subcutaneous tissue thickness was <20 mm.

The US evaluation of the liver can be performed using a low-frequency probe and the general findings of liver US can be assessed with no need for extensive training (Figure 1(d)) [46].

Two studies have specifically focused on the efficiency of pocket-sized US for assessing the liver. In the first study, 100 adults undergoing conventional abdominal US examinations for various indications were screened by POCUS immediately prior to conventional US. POCUS was only used to assess the presence or the absence of excess fat. Other liver disorders were not assessed. The investigators (conventional US: an experienced radiologist and POCUS: a general internist recently trained in the use of POCUS) were blinded to the results of the alternative imaging. Forty patients (40%) showed fatty infiltration of the liver on both conventional US and POCUS, and 49 (49%) were negative on both modalities. A consensus was reached in two out of the 11 remaining subjects whose results were initially discordant. The overall sensitivity and specificity of POCUS compared to conventional US were 91% and 88%, respectively [47]. These data were concordant with the study by Reily et al. showing that after a 20-minute teaching session, physicians are able to diagnose fatty liver infiltration with a positive predictive value of 94% and a negative predictive value of 96% [48]. Despite the fact that liver US is an easy to use and efficient tool allowing assessing the presence of NAFLD, its use is limited in severely obese patients and in patients with steatosis of less than 20-30% [42].

### 2.2. Lipohypertrophy

Lipohypertrophy (LH) occurs in the subcutaneous tissue as a result of the lipogenic effect of repeated insulin injections and repeated trauma induced by the needle [49, 50]. LH lesions are histologically characterized by decreased vascularity, fibrosis, and both hypertrophic and small neomitotic adipocytes. The risk factors include needle reuse, the absence of site rotation, a low level of education, the number of injections, and diabetes duration. Clinically, LH is characterized by thickened, “rubbery” tissue swelling and may be assessed by inspection and palpation. However, this method is poorly reliable with a high interobserver variation. Most studies suggest that insulin absorption from LH sites may be delayed and erratic [51], and the consequences are potentially dramatic with glycemic variability and an increased risk of severe hypoglycemia [52, 53]. The prevalence of LH varies considerably between the studies from 14.5% to 88%, reflecting diagnosis difficulties [51]. In 2013, Blanco et al. [54] have shown that 40% of patients with LH experienced unexplained hypoglycemia and 49% showed glycemic variability compared to only 5.9% and 6.5%, respectively, in patients without LH. These patients have increased insulin needs leading to an additional cost of €122 million in Spain. In China, the LH-related excess annual insulin consumption cost is estimated at $297 million [55]. Detecting LH is therefore crucial. The use of US could help to improve the diagnosis of LH.

The US findings of LH include the simplest subcutaneous hypertrophy, diffuse hyperechoic subcutaneous dystrophy, nodular hyperechoic dystrophy, focal and diffuse hyperechoic subcutis dystrophy, nodular hypoechoic subcutaneous dystrophy, subcutaneous atrophy, or complex multilayer dystrophy [56].

In an observational retrospective study, Bertuzzi et al. [57] have assessed 20 type 1 diabetes patients with LH by US using a linear probe (6-18 MHz). They have shown that the tissue affected by LH showed fibrotic changes (hyperechogenic) and interstitial edema (hypoechogenic). They have thus advised patients to avoid insulin injections in the LH areas seen on US. After 3 months, the HBA1c level was reduced from 7.87 ± 0.56% to 7.67 ± 0.52% (*p* = 0.029). In their study, LH areas showed at least three different aspects on US: an isohyperechogenic aspect with a predominant fibrotic component, an isoechogenic aspect with “large tangle” fibrotic components, and an isohypoechogenic aspect with no fibrotic components. No significant improvements in HbA1c were found in the control matched group in which LH was only clinically assessed through inspection and palpation. Thus, US can help to identify and characterize LH areas and could be useful to improve the glycemic control. A study has compared clinical and US examinations for the diagnosis of LH [58]. In this study, 103 patients, mainly with T2D treated with insulin for more than 2 years, were examined by 2 specialized nurses and then underwent US performed by a research associate trained by a certified radiologist. US identified subjects with LH significantly more frequently than the inspection or palpation (55% versus 72%; *p* < 0.0001). Among the subjects with LH lesions detected by US, 24% had lesions only detected by US. These findings show that US could be a promising tool to be used as an adjunct to palpation, but so far, no study has investigated whether US alone could detect LH independently of palpation.

### 2.3. Muscle

T2D facilitates the occurrence and progression of chronic complications such as diabetic neuropathy and sarcopenia. Diabetes accelerates the loss of muscle mass and strength over time, particularly in the lower extremities, which are associated with an increased risk of mortality in subjects with T2D [59–61]. DP have an altered body composition and a low musculoskeletal strength with a faster loss of knee extension strength compared to older NDS [62]. There is an age-related increased fatty infiltration of the midthigh skeletal muscle in men and women as shown by increases in intermuscular fat [63]. This fat infiltration worsens over 5 years in both men and women, regardless of weight changes and changes in subcutaneous adipose tissue in the thighs. Glucose fluctuations are also associated with a low muscle mass [64]. Thus, assessing and preserving the muscle mass is a critical point in these patients.

The quadriceps architecture that reflects the muscle mass (mainly by determining the thickness of the vastus intermedius, rectus, and anterior quadriceps) (Figure 1(f)) may be assessed using a high-frequency linear probe. The probe is positioned at half of the distance between the greater trochanter and the interarticular line of the knee, transversally to the muscle for measuring the thickness and cross-sectional area, and longitudinally for measuring the pennation angle (PA) in patients in prone position. Chiaramonte et al. [65] have shown the accuracy, precision, and repeatability of US in assessing the muscle architecture between physiatrists, radiologists, and general internists, and the quadriceps femoris muscle thickness assessed by US is already used as a parameter for assessing the nutritional risk that is more accurate than serum levels of prealbumin, albumin, or transferrin that may vary with the intravascular volume excess, infection, and inflammation [66].

Only one study [67] has assessed the reliability and applicability of quadriceps muscle architecture measurements in T2D patients. They have used a 10-13 MHz probe to assess the thickness of the rectus femoris (RF), vastus intermedius (VI), anterior quadriceps (sum of RF and VI), and the PA of the RF. T2D patients had neuropathy without osteoarticular injury and were older than 50. The PA of the RF was determined at the intersection between the muscle fascicles of the RF and the internal aponeurosis. Intra- and interrater analyses have shown a high to very high reliability between the three raters except for the PA.

Further studies are needed to precisely determine the role of US in the evaluation of the muscle mass and osteoarticular complications in DP, but it seems promising.

### 2.4. Gastroparesis

Gastroparesis is characterized by a delayed gastric emptying of solid food in the absence of a mechanical obstruction of the stomach, resulting in the cardinal symptoms of early satiety, postprandial fullness, nausea, vomiting, belching, and bloating [68]. It usually affects DP with other neuropathic diseases, affecting 30 to 50% of DP (T1D or T2D). Gastroparesis should be investigated because it induces a risk of stasis (full stomach) and aspiration upon anesthetic induction. Delayed gastric emptying and the resultant “full stomach” are the most important risk factors for perioperative regurgitation and aspiration, which remain common, disastrous complications associated with high morbidity and mortality in patients undergoing general anesthesia [69]. US examinations performed 2 h after ingesting a clear fluid or 6 h after a light meal using a low-frequency (2-5 MHz) curvilinear array probe from a Philips device (CX50) have shown that almost half of the T2D patients with a median diabetes duration of 6 years had a full stomach when the current preoperative fasting guidelines were followed [70]. Other studies have confirmed that US allows determining the gastric residual volume [71].

#### 2.4.1. Questions about Overdiagnosis

The question of overdiagnosis should be raised. Although the studies remain limited, the first data are quite reassuring. The rate of incidentalomas is lower in symptomatic patients (0.05%) and can reach up to 25% in asymptomatic patients [48].

## 3. Conclusion

POCUS is a diagnostic aid already used in many fields of internal medicine. Its use in diabetology is promising, but further studies are needed to confirm the data from the studies reported here. It allows improving the determination of the CV profile of DP but also allows monitoring the complications.
